# Breaking Barriers—The Intersection of AI and Assistive Technology in Autism Care: A Narrative Review

**DOI:** 10.3390/jpm14010041

**Published:** 2023-12-28

**Authors:** Antonio Iannone, Daniele Giansanti

**Affiliations:** 1 CREA, Italian National Research Body, Via Ardeatina, 546, 00178 Roma, Italy; 2 Centro Nazionale TISP, Istituto Superiore di Sanità; Viale Regina Elena 299, 00161 Roma, Italy

**Keywords:** assistive technology, accessibility, AAC, autism, AI, artificial intelligence

## Abstract

(Background) Autism increasingly requires a multidisciplinary approach that can effectively harmonize the realms of diagnosis and therapy, tailoring both to the individual. Assistive technologies (ATs) play an important role in this context and hold significant potential when integrated with artificial intelligence (AI). (Objective) The objective of this study is to analyze the state of integration of AI with ATs in autism through a review. (Methods) A review was conducted on PubMed and Scopus, applying a standard checklist and a qualification process. The outcome reported 22 studies, including 7 reviews. (Key Content and Findings) The results reveal an early yet promising interest in integrating AI into autism assistive technologies. Exciting developments are currently underway at the intersection of AI and robotics, as well as in the creation of wearable automated devices like smart glasses. These innovations offer substantial potential for enhancing communication, interaction, and social engagement for individuals with autism. Presently, researchers are prioritizing innovation over establishing a solid presence within the healthcare domain, where issues such as regulation and acceptance demand increased attention. (Conclusions) As the field continues to evolve, it becomes increasingly clear that AI will play a pivotal role in bridging various domains, and integrated ATs with AI are positioned to act as crucial connectors.

## 1. Introduction

### 1.1. Autism Diagnosis and Therapy

Autism, scientifically known as autism spectrum disorder (ASD), is a neurodevelopmental condition characterized by a wide range of challenges in social communication, language, behavior, and social interaction [1,2,3,4,5]. The manifestations of autism vary widely, giving rise to the concept of the “spectrum”, which includes individuals with mild to severe symptoms. Signs of autism can emerge from early childhood but are often identified in preschool or school age, when they become more evident. Symptoms include difficulty with verbal and nonverbal communication, difficulty interacting with others, repetitive and restricted interests and activities, and increased or decreased sensory sensitivity. To diagnose autism, a multidisciplinary approach is used [6,7,8]. Specialists, such as psychologists, child psychiatrists, and pediatricians, conduct interviews and observations to evaluate the individual’s behavior, language, social skills, and cognitive abilities. Diagnosis is often completed through structured questionnaires, developmental assessments, and assessments of communication skills [1,2,3]. In addition to behavioral assessments and questionnaires, genetic analysis can be an integral part of the diagnosis of autism since there is a genetic component to its etiology [2,3,4,5]. Blood tests and genetic tests can identify genetic abnormalities associated with autism [4]. Also, imaging can have a strategic role, as in the case of functional magnetic resonance [9], which is also integrated with AI [10,11]. Therapy is crucial in autism, providing specialized support to address the cognitive, communication, and behavioral challenges associated with the disorder. Through targeted therapeutic interventions, individuals with autism can develop social, communication, and adaptation skills, improving their quality of life and promoting greater inclusion in society [12,13,14,15,16]. Therapy represents an essential foundation for promoting the progress and well-being of people with autism. Multidisciplinary approaches in autism involve different professionals, such as psychologists, occupational therapists, speech therapists, and pediatricians, who collaborate to provide holistic treatment targeted to the specific needs of each individual with autism [12,14,15]. This synergy between experts contributes to a more complete, personalized, and effective intervention, addressing cognitive, communicative, and behavioral challenges in an integrated way and optimizing the progress and well-being of patients. Assistive technology (AT) tools provide personalized technological solutions for people with autism, helping them overcome communication barriers and adapt to their specific needs [16]. These tools amplify skills and improve independence, significantly contributing to the quality of life of people with autism.

### 1.2. Beyond Communication: The Versatility of Assistive Technology in Autism Care

Assistive technologies [17,18,19,20,21], ranging from robots [17] to sensors [18], particularly Augmentative and Alternative Communications (AAC) [19], play a fundamental role in improving the lives of people with autism by addressing the communication challenges that often accompany this disorder. For many people with autism, verbal communication can be a significant barrier. AAC [19] offers an alternative avenue, allowing these people to express themselves in ways that reflect their individual needs. This customization is critically important, as autism is an extremely heterogeneous disorder, and what works for one individual may not be as effective for another. One of the main benefits of AAC [19,20] is the reduction of frustration. The inability to communicate effectively can lead to high levels of anxiety and stress. AAC reduces this frustration by providing a means to express needs and desires, helping to improve mental health and interpersonal relationships. These technologies also have a significant impact on education. AAC can be used to support learning, helping students with autism develop language and cognitive skills [19]. Furthermore, they improve school inclusion, allowing students with autism to actively participate in educational activities. Another area in which AAC proves essential is improving social interactions. AAC facilitates communication and the establishment of meaningful relationships, which is often challenging for people with autism. These tools help people with autism participate more actively in conversations and social activities, improving the quality of their interactions. Independence is an important goal for many people with autism. AAC contributes to this goal by allowing people to communicate their needs and make autonomous decisions, promoting a greater level of autonomy in daily life. Overall, assistive technologies, such as AAC, are a valuable resource for people with autism. They enable them to overcome communication challenges, improve the quality of social interactions, support learning, and promote independence. These tools are fundamental in the field of autism, contributing significantly to the well-being and inclusion of these people in society.

### 1.3. AI’s Potential in Autism Assistive Technologies

Artificial intelligence (AI) could play a significant role in helping to personalize assistive technologies for individuals with autism [22,23]. From a future perspective, it could be capable of conducting a precise assessment of individual needs. AI, at least potentially, could analyze complex data, such as an individual’s behaviors and responses, to determine which tools and supports would be most suitable. This would mean that AI could contribute to designing solutions tailored to each individual, taking specific needs into account. Another potential of AI would be to adapt assistive technologies in real-time. This would mean that devices could automatically adjust settings based on user interactions and behaviors. For instance, if a person with autism were displaying signs of stress or frustration, AI could intervene to offer targeted support. Another important potential aspect of AI is machine learning. AI could learn from the user’s progress and challenges over time. This would mean that assistive technologies could continually improve their effectiveness, adapting to the evolving needs of individuals with autism. AI could also be capable of customizing the interfaces of assistive technologies to meet user preferences. This could make the tools more accessible and user-friendly, facilitating interaction and usage by the individual. Finally, AI could contribute to creating personalized learning and communication programs. These programs could take into account the individual’s skill level and specific progress, offering targeted support to help them develop their abilities. In summary, AI could play a key role in tailoring and optimizing assistive technologies for individuals with autism, contributing to improving their quality of life and fostering greater engagement and well-being.

### 1.4. Potential Emerging Questions

Key questions in personalizing assistive technologies with AI in ASD are emerging from the above:

- How can AI precisely assess individual needs for individuals with autism?
- In what ways can AI enable the real-time adaptation of assistive technologies?
- How does machine learning enhance the adaptability of assistive technologies over time?
- To what extent can AI customize interfaces for user preferences in assistive technologies?
- How can AI create personalized learning and communication programs for individuals with autism? What evidence exists regarding the impact of AI-driven personalization on the quality of life for individuals with autism?
- What ethical considerations are crucial when implementing AI in assistive technologies for individuals with autism?
- How can AI contribute to a more user-centered design approach in developing assistive technologies?

These questions suggest the need for a review.

### 1.5. Purpose of the Study

The purpose of this study is to explore the potential benefits and challenges associated with the integration of AI into assistive technologies for individuals with autism. By overviewing the ways in which AI can personalize and adapt these technologies to meet the specific needs of individuals on the autism spectrum, this article aims to shed light on the opportunities for improved support, communication, and quality of life for this community. Additionally, it seeks to highlight the importance of ongoing research and innovation in this field to ensure that individuals with autism receive the most effective and personalized assistance possible.

## 2. Methods

This review used the ANDJ standardized checklist designed for the narrative category of reviews [24]. The narrative review was performed based on targeted searches using specific composite keys on PubMed and Scopus.

The overview literature accompanying the main survey was conducted using both a qualification checklist and a qualification methodology based on proposed quality parameters described in [25] to decide the inclusion of the study in the overview.

### Algorithm Used in the Literature Overview

1. Set the search query to “defined search query”.
2. Conduct a targeted search on PubMed and Scopus using the search query from step 1.
3. Select studies published in peer-reviewed journals that focus on the field.
4. For each study, evaluate the following parameters:
  - N1: Is the rationale for the study in the introduction clear?
  - N2: Is the design of the work appropriate?
  - N3: Are the methods described clearly?
  - N4: Are the results presented clearly?
  - N5: Are the conclusions based on and justified by the results?
  - N6: Did the authors disclose all the conflicts of interest?
5. Assign a graded score to parameters N1–N5, ranging from 1 (minimum) to 5 (maximum).
6. For parameter N6, assign a binary assessment of “Yes” or “No” to indicate if the authors disclosed all the conflicts of interest.
7. Preselect studies that meet the following criteria:
  - Parameter N6 must be “Yes”.
  - Parameters N1–N5 must have a score greater than 3.
8. Include the preselected studies in the overview.

**Defined** **search** **query** **1.** *“self help devices”[MeSH Terms] OR (“self help”[All Fields] AND “devices”[All Fields]) OR “self help devices”[All Fields] OR (“assistive”[All Fields] AND “technology”[All Fields]) OR “assistive technology”[All Fields] OR (“augmentative”[All Fields] AND (“communicate”[All Fields] OR “communicated”[All Fields] OR “communicates”[All Fields] OR “communicating”[All Fields] OR “communication”[MeSH Terms] OR “communication”[All Fields] OR “communications”[All Fields] OR “communicative”[All Fields] OR “communicational”[All Fields] OR “communicatively”[All Fields] OR “communicativeness”[All Fields] OR “communicator”[All Fields] OR “communicator s”[All Fields] OR “communicators”[All Fields])) AND “autism”[Title/Abstract] AND (“artificial intelligence”[MeSH Terms] OR (“artificial”[All Fields] AND “intelligence”[All Fields]) OR “artificial intelligence”[All Fields] OR (“machine learning”[MeSH Terms] OR (“machine”[All Fields] AND “learning”[All Fields]) OR “machine learning”[All Fields]) OR (“deep learning”[MeSH Terms] OR (“deep”[All Fields] AND “learning”[All Fields]) OR “deep learning”[All Fields]) OR ((“neural”[All Fields] OR “neuralization”[All Fields] OR “neuralize”[All Fields] OR “neuralized”[All Fields] OR “neuralizes”[All Fields] OR “neuralizing”[All Fields] OR “neurally”[All Fields]) AND “nework”[All Fields]))*

We applied the defined algorithm for the selection of the articles. In particular, after applying points 3 and 7, we’ve pinpointed a total of 22 studies [26,27,28,29,30,31,32,33,34,35,36,37,38,39,40,41,42,43,44,45,46,47]. It’s interesting to highlight that this list precisely matches the number of studies detected in PubMed after excluding one retraction. Scopus, it’s important to mention, contained a few conference papers that our algorithm chose to exclude for specific reasons. Out of these 22 studies, seven are comprehensive reviews, encompassing both systematic and non-systematic ones.

The remaining 15 studies are a mix of scientific articles and various other papers.

## 3. Results

The results have been organized into two parts and presented editorially through two main paragraphs.

In the first part (Section 3.1), a thorough examination is dedicated to the findings extracted from reviews and systematic reviews. Researchers can delve into distilled and structured insights at the crossroads of AI and assistive technologies (ATs) in the context of autism. Reviews and systematic reviews are distinguished from other articles as they offer a broader perspective, functioning as filters that distill the wealth of existing research. This aids researchers in identifying common themes, emerging patterns, and gaps in current knowledge.

The second part (Section 3.2) broadens the research’s scope by delving into the outcomes of the remaining studies. This approach captures a more diverse array of perspectives concerning the intersection of AI and ATs in autism. Through a critical examination of these remaining studies, researchers can incorporate various viewpoints and alternative methodologies and potentially discover novel or previously overlooked insights. This holistic understanding of the research landscape promotes a more balanced and nuanced interpretation of the subject, reducing the risk of overlooking valuable contributions to the field.

In essence, this dual approach enhances both the depth and breadth of the research, resulting in a more robust and holistic analysis.

### 3.1. In-Depth Analysis of the Detected Reviews: A Comprehensive Overview

#### 3.1.1. Analysis in Details

Seven review studies have been detected facing the intersection of AI and ATs in autism.

The review proposed by Muthu et al. [26] emphasizes the significant impact of assistive technology for differently-abled individuals and older adults, covering rehabilitative, adaptive, and assistive devices. It discusses the applications, challenges, and potential for enhancing daily life, with a special focus on AI-powered technologies with reference to autism. The study sheds light on the pros and cons of these technologies, offering valuable insights for rehabilitation engineering.

Focusing on mental disorders with childhood onset, the review by Datta Barua et al. [27] explores the co-morbidity of neurodevelopmental and mental health disorders. It highlights the role of AI-assisted tools in addressing learning challenges in individuals with neurodevelopmental disorders. The review points to the potential of AI tools for improving social interaction and personalized education.

Alabdulkareem et al. [28] delve into the use of interactive robots in autism therapy, utilizing AI technologies. The study analyzes trends in research, showing a significant increase in journal publications in the field, driven by advances in artificial intelligence techniques and machine learning. This highlights the growing role of AI in robot-assisted autism therapy.

Ur Rehman et al. [29] explore in their study the impact of mobile applications, particularly those utilizing AI technologies, on the lives of individuals with autism spectrum disorder (ASD). The study identifies features of highly-rated apps, offering recommendations for enhancing existing applications with AI. Results suggest the potential for progress tracking, personalized content delivery, automated reasoning, image recognition, and natural language processing (NLP) in these AI-powered apps.

Di Pietro et al. [30] focused on computer-assisted and robot-assisted therapies for children with autism spectrum disorder. It aims to identify the types of information technology platforms being used, the professions involved, the outcomes being evaluated, and the benefits to children with autism, with a keen eye on AI-enhanced interventions. The review highlights the promise of these AI-powered interventions while also stressing the need for further research.

Den Brok et al., in their systematic review [31], investigate the use of self-controlled technologies for persons with autism spectrum disorder and intellectual disabilities, some of which leverage AI. The results show that these technologies facilitate the learning of daily living skills and cognitive concepts, with a particular emphasis on AI-powered features. Advanced technologies, such as virtual reality, are effective for learning cognitive concepts. However, more research is needed to assess generalization and the role of AI in effectiveness. Billard et al. [32] dealt with the outcome of a project focused on robotics, the Robota project. This project employs humanoid robots in behavioral studies with low-functioning children with autism, with a focus on the technological aspects, including AI. The review discusses the technological developments and outcomes of these studies, emphasizing the potential for using imitator robots to assess and teach coordinated behaviors and the role of AI in enhancing these interventions. This work informs the future development of robots for children with complex developmental disabilities, incorporating AI-driven innovations.

We have also detected in Table 1 the key elements/points highlighting the intersection of AI with ATs across the studies, showcasing AI’s contribution to enhancing assistive technologies for various applications in healthcare, education, and therapy.

It appears that the studies do not extensively cover the limitations and bottlenecks of AI within assistive technologies. The emphasis in these reviews is primarily on the benefits and potential of AI in various applications, while limitations and challenges are not explicitly addressed.

#### 3.1.2. Key Findings

Collectively, the body of research from various studies [26,27,28,29,30,31,32] underscores the profound impact and diverse applications of AI within ATs, especially for individuals with neurodevelopmental disorders like autism. The studies collectively reveal a promising landscape where AI-driven solutions are actively contributing to the enhancement of rehabilitation, independence, and overall well-being. These solutions address a spectrum of challenges, ranging from physical impairments and mobility issues to personalized education for individuals with neurodevelopmental disorders. In the realm of autism therapy, the use of interactive robots equipped with AI is a notable trend, reflecting a growing recognition of AI’s potential in improving social interaction and engagement. The observed expansion of research in this area attests to the increasing importance and applicability of AI techniques and machine learning within autism therapy.

Additionally, the collective effort in identifying highly-rated mobile apps for individuals with ASD using AI technologies and the subsequent recommendations for enhancing existing applications exemplify a practical approach to leveraging AI for personalized support. This emphasizes the ongoing commitment to tailoring AI-driven tools to the specific needs of individuals with ASD. The exploration of AI-driven computer-assisted and robot-assisted therapies further extends the application of AI into educational contexts for children with autism. The studies shed light on the identification of AI platforms, the involvement of various professions, and the outcomes of social skills teaching, collectively enriching our understanding of how AI can be effectively integrated into educational interventions. Moreover, the use of AI-powered self-controlled technologies for individuals with autism and intellectual disabilities emphasizes the potential of AI to facilitate learning in various domains, including daily living skills and cognitive concepts. The collective findings highlight the adaptability of AI in catering to diverse learning needs within the neurodevelopmental disorder spectrum. In the context of humanoid robots assisting low-functioning children with autism, the studies collectively delve into the intricacies of using AI to assess imitation ability and teach coordinated behaviors. This offers insights into both the challenges and possibilities associated with optimizing the role of AI within humanoid robots for targeted support. Common methodologies across these studies include an interdisciplinary approach, demonstrating collaboration across various professions to integrate AI effectively into ATs. The shared emphasis on developing AI-driven tools for personalized education and support reflects a collective commitment to tailoring interventions to individual needs. However, the challenges identified collectively, such as the ethical considerations associated with privacy, responsible AI use, and the need for interdisciplinary collaboration, underscore the complexities of integrating AI into ATs. These challenges collectively call for a nuanced and thoughtful approach to ensure the responsible and effective use of AI in enhancing the lives of individuals with neurodevelopmental disorders. In summary, the collective view across these studies paints a comprehensive picture of AI’s transformative potential in the realm of assistive technologies, showcasing its versatility and adaptability in addressing diverse challenges and providing personalized support for individuals with neurodevelopmental disorders.

### 3.2. In-Depth Analysis of the Detected Articles: A Comprehensive Overview

#### 3.2.1. Analysis in Details

Fifteen studies have been detected [33,34,35,36,37,38,39,40,41,42,43,44,45,46,47] in this part of the overview. In some articles, the intersection of AI and ATs is directly faced [33,34,35,36,37,38,39,40,41,42,43,45,46,47], while in others, it is faced more with an in-perspective overview [44].

Silvera Tawill et al. [33] explore the use of socially-assistive robots, incorporating AI, to support children with autism. The study identifies barriers to implementation and teachers’ and therapists’ expectations, highlighting the potential of AI-driven robots for teaching support.

Deng et al. [34] introduce a sensory management recommendation system that relies on AI techniques to assist children with ASD in dealing with sensory issues. The system uses sensor fusion and machine learning to identify distractions, anxious situations, and their causes, enabling more effective interventions.

Wan et al. [35] propose an AI-based system for improving emotion recognition in Chinese children with ASD. The system incorporates deep learning algorithms for facial expression recognition and attention analysis, demonstrating its potential in AI-assisted therapies.

Kumar et al. [36] examine the automation of ASD diagnosis using machine learning techniques. The study leverages AI to analyze a dataset of 701 samples, aiming to develop models that can assist in diagnosing ASD automatically.

Jain et al. [37] utilize supervised machine-learning algorithms to model user engagement in long-term, AI-driven, socially-assistive robot interventions for children with ASD. AI models achieve high accuracy in recognizing and responding to user engagement, enhancing human-robot interactions.

Keshav et al. [38] correlate student performance on the Empowered Brain platform with clinical measures of ADHD, demonstrating that AI-driven technologies can aid in monitoring and managing symptoms of co-occurring conditions in students with ASD.

Vahabzadeh et al. [39] explore the feasibility and efficacy of Empowered Brain, an AI-driven smartglasses intervention for students with ASD. It demonstrates the potential of AI in improving socio-emotional behaviors, highlighting its impact in a school-based intervention.

Cooper et al. [40] introduce an AAC software program with an embedded artificial conversational agent, named Alex. The software is designed to assist children with autism who use augmentative and alternative communication (AAC) aids. Alex utilizes symbols and images, can be personalized by therapists, and does not require specialized computer skills. The software emphasizes customization, interoperability, personalization, and considerations for motor skills.

Huijnen et al. [41] focus on the roles, strengths, and challenges of robot KASPAR in interventions for children with ASD, including its use of AI components such as personalization and consistent application of actions.

Keshav et al. [42] assess the tolerability and usability of the Brain Power Autism System (BPAS), which integrates AI and smartglasses. The outcome shows that AI-driven wearable technology is well tolerated and usable by individuals with ASD, emphasizing its role as an assistive technology.

Linstead et al. [43] investigate the influence of treatment intensity and duration on learning in children with autism who are receiving Applied Behavior Analysis (ABA) services. The study assesses the impact of these treatment variables on various domains, such as academic, adaptive, cognitive, executive function, language, motor, play, and social skills. The findings highlight the importance of treatment dosage and provide insights into its varied effects across different domains and the usefulness of AI in perspective.

Desideri et al. [44] explore the potential of a humanoid robot to enhance educational interventions for children with autism spectrum disorders (ASD). Preliminary results indicate that interacting with a humanoid robot can facilitate engagement and goal achievement in educational activities. This highlights the role of advanced technology and AI in improving the effectiveness of educational interventions for children with ASD.

Huijnen et al. [45] aim to practically implement robots, specifically robot KASPAR, into current education and therapy interventions for children with ASD. The study involves focus groups and co-creation sessions with professionals and adults with ASD. It results in requirements for robot-assisted interventions, a template for describing robot interventions, and the generation of new intervention ideas, emphasizing the practical application of robots with AI capabilities in autism therapy and education.

Bekele et al. [46] investigate an AI-driven robot-mediated system to administer joint attention prompts to children with ASD, demonstrating the potential of AI to enhance engagement and learning in educational activities for these children.

Williams et al., in a multicenter study [47], use a computer program with speech synthesizer software and a “virtual” head to investigate audio-visual integration in children with ASD. AI-like systems facilitate speech recognition and training, showcasing the role of AI in improving speech-related skills in individuals with ASD.

For these scientific articles, we have also detected in Table 2 the key points/elements highlighting the intersection of AI with ATs across the studies, showcasing AI’s contribution to enhancing assistive technologies for various applications in healthcare, education, and therapy.

#### 3.2.2. Key Findings

The collection of studies [33,34,35,36,37,38,39,40,41,42,43,44,45,46,47] collectively sheds light on the promising intersection of artificial intelligence (AI) and autism assistive technologies [33,34,35,36,37,38,39,40,41,42,43,44,45,46,47]. The research landscape reflects a concerted effort to harness AI’s capabilities for diverse applications catering to the unique needs of individuals on the autism spectrum.

A recurring theme in these studies is the exploration of AI-driven devices, such as socially-assistive robots, smartglasses, and recommendation systems, to enhance various facets of support for individuals with ASD. This includes teaching support, sensory management, emotion recognition, and even the automation of ASD diagnosis. The integration of AI into AAC software, featuring artificial conversational agents, stands out as a noteworthy endeavor to enhance communication for children with autism.

Common methodologies across these studies include the prevalent use of AI and machine learning techniques. Researchers are leveraging these advanced technologies to develop recommendation systems, models for recognizing and responding to user engagement, and diagnostic tools. Additionally, the implementation of AI-driven devices, particularly in educational interventions, emerges as a consistent approach to improving engagement and learning outcomes for individuals with ASD.

However, amidst the optimism, several challenges are evident. The diversity within the autism spectrum poses a substantial hurdle, requiring nuanced approaches that account for individualized needs. Ethical considerations, ranging from the tolerability and usability of AI-driven devices to broader privacy concerns, are inherent in the integration of AI into assistive technologies. The regulatory landscape and societal acceptance of these innovations within the healthcare domain represent additional challenges that demand careful navigation.

The practical implementation of humanoid robots in education and therapy interventions introduces complexities, with studies highlighting both the strengths and challenges associated with these AI-equipped robots. Moreover, the studies collectively underscore the imperative to strike a balance between pushing the boundaries of technological innovation and addressing the practical and ethical considerations essential for the successful integration of AI into autism assistive technologies.

In essence, this body of research paints a dynamic picture of the evolving relationship between AI and autism assistive technologies. The studies not only showcase the potential of AI to revolutionize support for individuals with ASD but also illuminate the path forward, emphasizing the need for a holistic and ethically grounded approach as these technologies continue to play an increasingly significant role in the lives of those on the autism spectrum.

## 4. Discussion

The discussion is structured into two parts, which are editorially translated into five paragraphs.

The initial part reported in Section 4.1, sets the stage, delving into the dissemination trends within this field. We examine these trends in contrast to the broader, overarching categories of ATs, which include AAC, both in a general context and, more specifically, within the realm of autism. The second part (Section 4.2, Section 4.3, Section 4.4 and Section 4.5) delves into: (a) a detailed discussion of the key findings that have emerged in the study results, paying careful attention to the distinctive characteristics of autism. (b) Analyze the limitations and bottlenecks that have come to light within the reviewed studies.

### 4.1. Numerical Trends in Assistive Technologies for Autism

**Figure 1:**
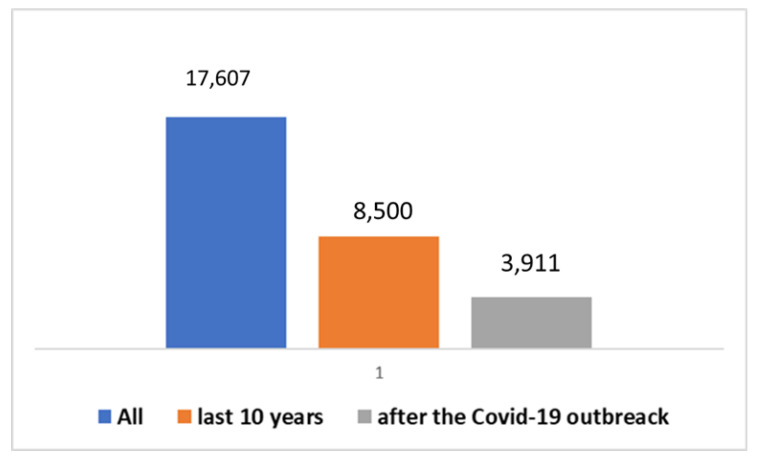
Trends in the studies on ATs (including AAC) over time.

**Figure 2:**
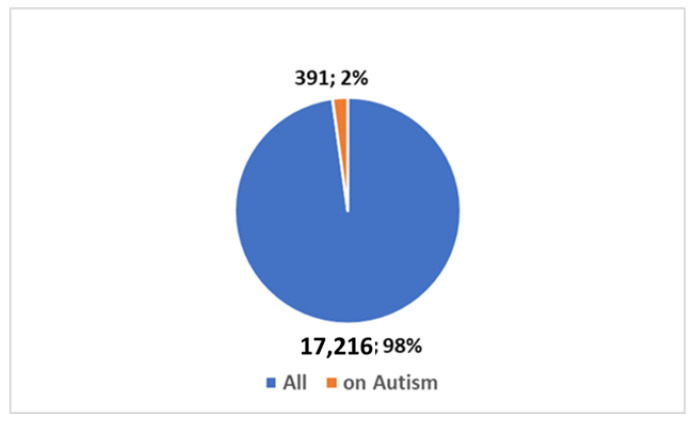
Percentage of studies on AT (including AAC) on autism compared to the total.

**Figure 3:**
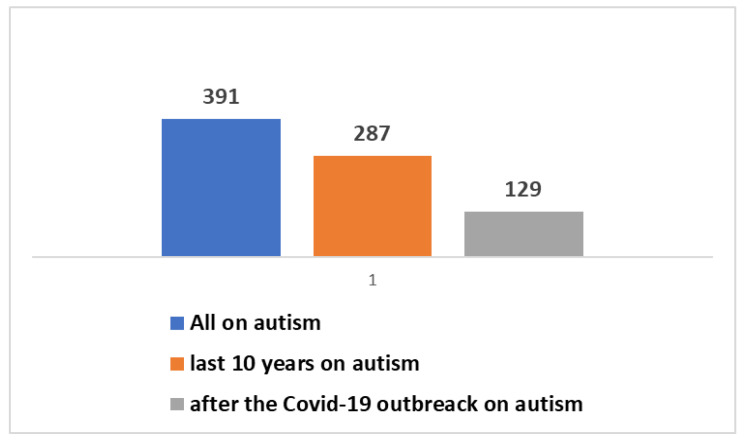
Scientific production of studies dedicated to AT (including AAC) on autism.

**Figure 4:**
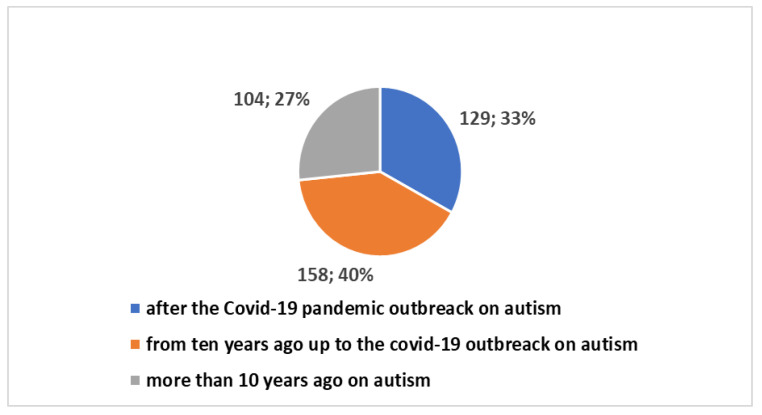
Percentage of studies dedicated to AT (including AAC) on autism over time.

**Figure 5:**
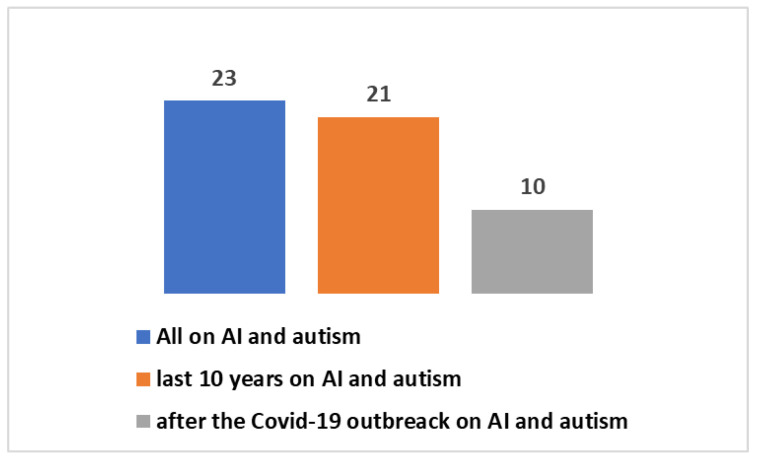
Studies on AI applied to ATs (including AACs) dedicated to autism.

It is valuable to delve into the analysis of scientific publication trends within this specific field. The key insights encapsulated in Box 1 have been utilized in research conducted on the PubMed platform. Scientific publications pertaining to AAC, as well as other assistive technologies, trace their origins back to the year 1946. In a broader context, as depicted in Figure 1, a cumulative total of 17,607 studies have been brought forth to the scientific community. Notably, a substantial 8500 of these have emerged within the last decade, with a further 3911 publications emerging since the onset of the global COVID-19 pandemic. A significant proportion of these studies, roughly 39%, have seen the light of day in the most recent decade, and particularly noteworthy is the surge in publications following the emergence of the COVID-19 pandemic, accounting for approximately 19% of the total.. The graphical representation in Figure 2 provides a visual insight into the fact that a mere 2% of these studies are centered around the subject of autism. Zooming in on the domain of autism, the data depicted in Figure 3 and Figure 4 reveal that, since 1992, a remarkable 391 studies have been produced on this subject. A considerable majority of these studies, (73%), have been published within the last decade. Furthermore, a significant 129 studies have been generated in the wake of the COVID-19 pandemic, constituting 33% of the total in this time frame. However, when we narrow our focus even further and zero in on those studies that specifically explore the intersection of autism and artificial intelligence (AI), the number is substantially reduced. In its entirety, only 23 studies have been published in this area. Out of these, 10 have emerged in the aftermath of the COVID-19 pandemic, while 21 have surfaced within the last ten years (as presented in Figure 5).

Box 1. **The proposed composite keys.**

*“self help devices”[MeSH Terms] OR (“self help”[All Fields] AND “devices”[All Fields]) OR “self help devices”[All Fields] OR (“assistive”[All Fields] AND “technology”[All Fields]) OR “assistive technology”[All Fields] OR (“augmentative”[All Fields] AND (“communicate”[All Fields] OR “communicated”[All Fields] OR “communicates”[All Fields] OR “communicating”[All Fields] OR “communication”[MeSH Terms] OR “communication”[All Fields] OR “communications”[All Fields] OR “communicative”[All Fields] OR “communicational”[All Fields] OR “communicatively”[All Fields] OR “communicativeness”[All Fields] OR “communicator”[All Fields] OR “communicator s”[All Fields] OR “communicators”[All Fields]))*

 

*(“self help devices”[MeSH Terms] OR (“self help”[All Fields] AND “devices”[All Fields]) OR “self help devices”[All Fields] OR (“assistive”[All Fields] AND “technology”[All Fields]) OR “assistive technology”[All Fields] OR (“augmentative”[All Fields] AND (“communicate”[All Fields] OR “communicated”[All Fields] OR “communicates”[All Fields] OR “communicating”[All Fields] OR “communication”[MeSH Terms] OR “communication”[All Fields] OR “communications”[All Fields] OR “communicative”[All Fields] OR “communicational”[All Fields] OR “communicatively”[All Fields] OR “communicativeness”[All Fields] OR “communicator”[All Fields] OR “communicator s”[All Fields] OR “communicators”[All Fields]))) AND “autism”[Title/Abstract]*

 

*(“self help devices”[MeSH Terms] OR (“self help”[All Fields] AND “devices”[All Fields]) OR “self help devices”[All Fields] OR (“assistive”[All Fields] AND “technology”[All Fields]) OR “assistive technology”[All Fields] OR (“augmentative”[All Fields] AND (“communicate”[All Fields] OR “communicated”[All Fields] OR “communicates”[All Fields] OR “communicating”[All Fields] OR “communication”[MeSH Terms] OR “communication”[All Fields] OR “communications”[All Fields] OR “communicative”[All Fields] OR “communicational”[All Fields] OR “communicatively”[All Fields] OR “communicativeness”[All Fields] OR “communicator”[All Fields] OR “communicator s”[All Fields] OR “communicators”[All Fields]))) AND “autism”[Title/Abstract] AND (“artificial intelligence”[MeSH Terms] OR (“artificial”[All Fields] AND “intelligence”[All Fields]) OR “artificial intelligence”[All Fields] OR (“machine learning”[MeSH Terms] OR (“machine”[All Fields] AND “learning”[All Fields]) OR “machine learning”[All Fields]) OR (“deep learning”[MeSH Terms] OR (“deep”[All Fields] AND “learning”[All Fields]) OR “deep learning”[All Fields]) OR ((“neural”[All Fields] OR “neuralization”[All Fields] OR “neuralize”[All Fields] OR “neuralized”[All Fields] OR “neuralizes”[All Fields] OR “neuralizing”[All Fields] OR “neurally”[All Fields]) AND “nework”[All Fields]))*

### 4.2. Interpretation of Results: Findings, Problems

Autism, as a neurodevelopmental disorder, affects social behavior, communication, and interaction. It manifests itself with difficulties in understanding other people’s emotions, in verbal and non-verbal communication, in restricted interest in certain topics, and in the repetitiveness of behaviors and routines. Each autistic individual is unique in his or her characteristics and level of functioning [48]. This uniqueness is reflected in the difficulty of diagnosis and therapy [49,50], which requires a multi-faceted approach from different medical disciplines, and in treatment that increasingly highlights the need for personalized medicine dedicated to autism [51,52].

**Figure 6:**
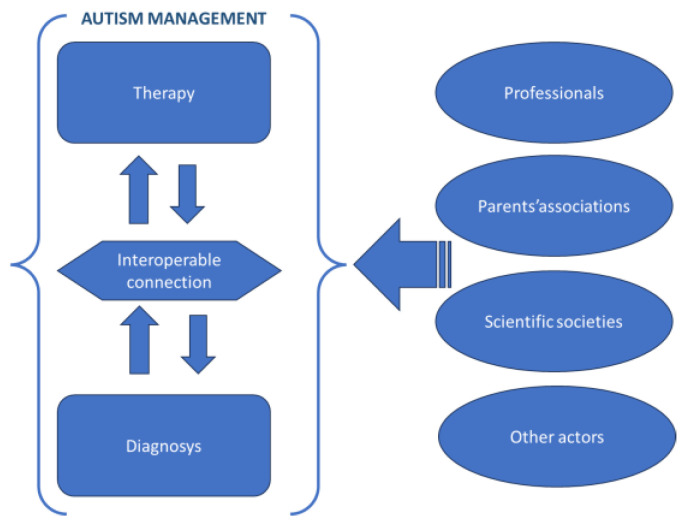
Autism: interoperability between the therapy and the diagnosis domains.

It can unequivocally be asserted that a sound approach to addressing autism hinges upon the seamless interplay between the domains of diagnosis and therapy, encompassing a multitude of key players, ranging from parental associations and diverse professionals to scientific organizations (Figure 6):

- If we focus on the autism diagnosis we can affirm that among the important activities in diagnosis we find [49,50]: -Observation and Interviews: -Physical Exam and Medical History: -Developmental Assessment and Screening: -Psychological and Psychomotor Evaluation: -Assessment of Social Behavior and Social Interactions: -Language and Communication Assessment: -Sensory Assessment-Functional Behavior Assessment. -Genetic, metabolic, biochemical, immunological, neurobiologal assessments-Environmental factors. -Medical Imaging assessment. There are various therapies and interventions used in the treatment of ASD. These therapies aim to address the unique challenges and needs of individuals with autism. Therapies may include medications.
- If we focus on the autism therapy we can affirm that some of the most commonly used non medication therapies include [51]: -Behavioral Therapies: -Communication and Speech Therapies. -Speech-Language Therapy-Occupational Therapy. -Social Skills Training-Sensory Integration Therapy. -Educational Interventions-Medication. -Alternative and Complementary Therapies. It’s important to note that the choice of therapy or intervention depends on the individual’s specific needs, strengths, and challenges [52,53]. A comprehensive and individualized treatment plan is often the most effective approach, and it should be developed in consultation with healthcare professionals, including speech therapists, occupational therapists, and behavioral specialists, to provide the best possible support for individuals with autism. There are also available programs that provide training and support for parents and caregivers to help them better understand and manage the challenges associated with autism.

ATs (including AAC) devices occupy a central and strategic position in a multitude of non-medical therapies, as highlighted. However, adhering to the expansive framework established by the WHO [54], which encompasses AT processes and related services, including telemedicine, it becomes evident that ATs possess considerable untapped potential, even within the realm of diagnostic activities.

Our review, through two perspectives (one focused on reviews and the other on scientific articles), has addressed the introduction of AI in assistive technologies for autism.

The initial perspective, cantered on comprehensive reviews, underscores a steadily escalating integration of artificial intelligence (AI) within assistive technologies (ATs) for autism, spanning across fields like robotics [27,30,32], applications [29], and, in a broader context, automated machines such as computers and ICT devices [26,27,28,29,30,31,32]. This integration is specifically geared towards the enhancement of communication, interaction, and, most importantly, the overall social development of autistic children. Notably, in the case of mobile applications, a set of valuable recommendations has been proposed as well [29].

The second perspective consistently underscores a fascinating and rapidly growing integration of AI with robotics in the context of autism [33,38,41,44,45,46]. This integration serves as vital support for enhancing communication, interaction, and social engagement. Furthermore, the strategic aspect of therapy dosage in the realm of autism has been explored across various domains [36]. There’s a burgeoning interest in the deployment of customized wearable devices, such as smart glasses [39,42], designed to improve socio-emotional behaviors in students with autism spectrum disorder (ASD), with their tolerability also undergoing assessment.

Across all of these research works, as seen in the perspective on reviews, AI is consistently addressed within the domain of automated machines, including computers and ICT devices [33,34,35,36,37,38,39,40,41,42,43,44,45,46,47]. In a particular study [36], as we had hoped for, the use of assistive technologies (ATs) in the field of diagnosis is highlighted, specifically the automation of ASD diagnosis through the application of machine learning techniques.

What emerges from the review is undoubtedly an early-phase landscape, particularly highlighted by the modest numbers evident at this stage in the field. Consequently, as expected, researchers are currently dedicating relatively little attention to aspects related to integration with the health domain, such as regulatory and consent issues.

Furthermore, it doesn’t seem that there is a strong focus on personalized medicine within the realm of AI in assistive technologies. Personalized medicine, also known as precision medicine or personalized medicine, could represent an innovative approach in the field of autism [55]. This approach would carefully consider individual differences, including genetics, lifestyle, and environment, with the aim of personalizing disease prevention, diagnosis, and treatment with the aim of maximizing therapeutic efficacy and minimizing side effects [56,57,58], while also integrating with AI [59,60]. In the specific context of autism, personalized medicine could seek to adapt treatments based on the specific genetic and biological characteristics of each individual suffering from ASD [61]. This could mean identifying specific subtypes of autism based on genetic, biochemical, and neurophysiological markers. This customization would allow for a more accurate diagnosis and personalized assessment of each patient’s clinical picture, helping to identify the most suitable and effective treatments [62,63,64].

**Figure 7:**
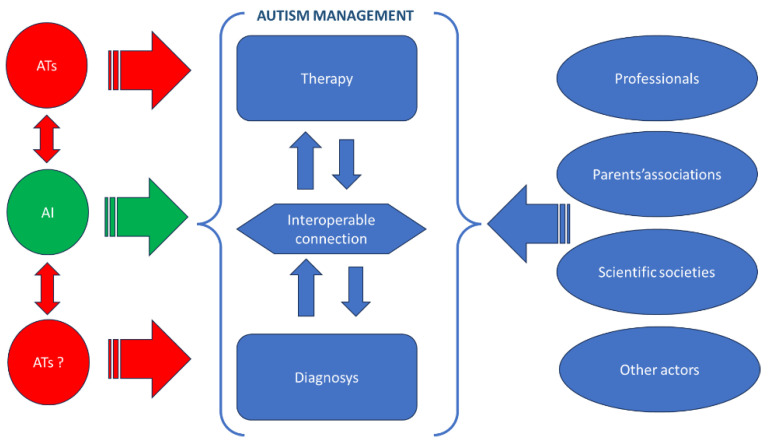
Autism: the potential mediator role of the AI and of the ATs.

It will unquestionably be imperative to exert additional efforts in these directions. Undoubtedly, AI is poised to play an ever more pivotal role in interconnecting diverse realms, and the integration of ATs with AI stands to assume an increasingly vital role as a connector of paramount importance (Figure 7).

### 4.3. Contextualizing Our Study: A Comparative Analysis with Diverse AI Applications in Autism Interventions

AI’s impact on autism research is substantial, with a growing interest in its applications [65,66,67]. From diagnostics to IoT integrations, AI’s transformative influence spans various healthcare facets, marking a remarkable evolution [68,69]. Machine learning (ML) and deep learning, especially in neural networks, play pivotal roles in addressing autism spectrum disorder (ASD) challenges [65]. ML excels in early ASD detection via behavioral and physiological data analysis, while predictive modeling tailors support strategies [66]. Naturalistic behavioral analysis, powered by computer vision and ML, informs interventions by decoding subtle cues [69]. Deep learning contributes to understanding communication challenges and identifying genetic markers associated with autism [65]. AI promises transformative potential in ASD research, from early detection to personalized interventions, exemplifying technology’s capacity to improve lives [65,66,67,68,69].

In [70], 11 systematic reviews [28,71,72,73,74,75,76,77,78,79,80] focused on the impact of AI on autism. Collectively, these reviews tell a compelling story of AI emerging as a powerful ally in autism research. Themes explored include precision psychiatry [71], virtual reality-based techniques for health improvement [72], bibliometric analysis of AI in autism treatment [73], hybridization of medical tests [74], triage and priority-based healthcare diagnosis [75], mobile and wearable AI in child and adolescent psychiatry [76], robot-assisted therapy [28], machine-learning models in behavioral assessment [77], deep learning in psychiatric disorders classification [78], the impact of technology on ASD [79], and deep learning in neurology [80]. Each systematic review contributes to a nuanced exploration of AI within the realm of autism research, shedding light on technology’s intersections with neurodevelopmental disorders. These reviews collectively underscore how AI is becoming integral in understanding and supporting individuals on the autism spectrum, offering diverse insights into tailored interventions, holistic well-being, diagnostic strategies, and advancements in neurology. Some of these themes have connections with assistive technologies (ATs). The proposed overview can serve as both a valuable contribution and a mediator and connector between some of these fields.

### 4.4. Reflections on the Limitations

The review indirectly highlights that integration of AI with assistive technologies in the context of autism presents several challenges that merit careful consideration. Firstly, the inherent diversity within the autism spectrum poses a significant limitation. The spectrum encompasses a broad range of characteristics, making it difficult to develop AI solutions that adequately address the unique needs of each individual. A one-size-fits-all approach may fall short in providing meaningful support. Moreover, the highly individualized nature of autism complicates the effectiveness of AI interventions. Each person with autism has distinct preferences, strengths, and challenges that evolve over time. AI technologies may struggle to keep pace with these individualized requirements, potentially limiting their utility. Ethical considerations also loom large in the integration of AI. The collection and analysis of personal information to tailor interventions raises concerns about privacy, consent, and the potential for data misuse. Striking a balance between leveraging data for customization and respecting ethical boundaries is crucial. A fundamental aspect of autism support is human connection and empathetic understanding. AI, by its very nature, lacks the ability to establish genuine emotional connections. This deficit in emotional support may impede the effectiveness of AI-based interventions, particularly for individuals who require a more empathetic touch. Additionally, user acceptance and comfort pose significant challenges. Individuals with autism may face difficulties adapting to or feeling comfortable with AI technologies. Overcoming resistance and ensuring user comfort are paramount to the successful integration of AI with assistive technologies. Overall, while the integration of AI with AT holds promise, navigating the limitations requires a nuanced approach. Addressing the diversity within the spectrum, recognizing the individualized nature of autism, and upholding ethical standards are essential for the meaningful and ethical use of AI in supporting individuals with autism.

### 4.5. Reflection on the Broader Implications

The review also shows that the integration of AI with ATs for individuals with autism introduces broader implications that encompass issues of bias, ethics, and cybersecurity. Concerns related to bias arise from the potential replication of societal biases within the AI algorithms. If the training data used is not representative or contains inherent biases, the AI systems may inadvertently perpetuate stereotypes and fail to address the diverse needs of individuals on the autism spectrum. Ethical considerations become paramount, particularly concerning privacy and informed consent. The customization of interventions based on personal data necessitates a clear understanding and explicit consent from individuals with autism and their caregivers. Ensuring transparency in decision-making processes and providing individuals with the ability to comprehend and challenge those decisions are ethical imperatives. Equity and accessibility issues emerge as the integration of AI may not guarantee equal access to interventions. This raises ethical concerns about the potential exacerbation of existing disparities, emphasizing the need for ethical considerations that ensure the inclusivity and accessibility of AI benefits for all individuals with autism. Turning to cybersecurity, the sensitive nature of the data involved in autism support systems becomes a focal point. The risk of cyberattacks targeting personal information, communication patterns, and behavioral data underscores the importance of robust data security measures to safeguard the privacy and well-being of individuals with autism. Moreover, vulnerabilities to malicious exploitation of AI systems need careful attention. Tampering with interventions, manipulating data, or using AI tools to harm individuals with autism are potential risks that demand proactive measures to secure AI technologies and prevent exploitation. The interconnected nature of AI and AT systems introduces cybersecurity challenges. A breach in one system could have cascading effects on others, potentially compromising the well-being and privacy of individuals with autism. Establishing a secure and resilient infrastructure becomes imperative to mitigate these interconnected cybersecurity risks. Overall, while the integration of AI with assistive technologies holds promise for individuals with autism, addressing biases, upholding ethical standards, and ensuring robust cybersecurity measures are critical for the responsible and beneficial use of AI in enhancing the lives of those on the autism spectrum. Striking a delicate balance between innovation and ethical considerations is paramount to navigating these complex implications.

## 5. Brief Summary and Conclusions

### 5.1. Brief Summary

The amalgamation of findings from 22 studies, encompassing 7 reviews, underscores a burgeoning interest in the integration of AI into autism assistive technologies. The current landscape is marked by promising developments at the intersection of AI and robotics, as well as the creation of wearable automated devices like smart glasses. These technological innovations are poised to significantly improve communication, interaction, and social engagement for individuals with autism, offering a glimpse into a future where AI plays a pivotal role in supporting neurodiversity.

However, as the field progresses, it becomes evident that the emphasis on innovation currently outweighs the establishment of a solid presence within the healthcare domain. Critical issues such as regulation and societal acceptance are demanding increased attention. This underscores the need for a delicate balance between pushing the boundaries of technological advancement and addressing the practicalities of integrating these innovations into mainstream healthcare practices. Despite the exciting prospects, limitations exist on this path towards integrating AI into autism assistive technologies. The diversity within the autism spectrum poses a challenge, as individualized needs vary widely. Ethical concerns, including those related to privacy and data security, emerge as critical considerations that must be carefully navigated. Furthermore, the potential absence of a human touch in AI interventions raises questions about user acceptance, particularly among individuals with autism who may require a more empathetic and personalized approach.

Looking at the broader implications, the innovative fusion of AI with autism assistive technologies opens doors to transformative possibilities. It holds the potential to bridge various domains and act as a crucial connector, facilitating communication and support for individuals with autism. However, as this field evolves, it is imperative to address ethical considerations, establish robust regulatory frameworks, and ensure that these technological advancements are accessible and inclusive for all individuals on the autism spectrum. In navigating this complex landscape, the role of AI in fostering connectivity and support for neurodiverse communities becomes increasingly evident.

### 5.2. Conclusions

In conclusion, our review highlights an early but promising interest in the integration of artificial intelligence into autism assistive technologies although not without significant problems to face. Particularly fascinating developments are unfolding in the fusion of AI with robotics and the creation of wearable automated devices, such as smart glasses. These advancements hold exciting potential for enhancing communication, interaction, and social activities for individuals with autism. Currently, researchers are dedicating more effort to development than to the establishment of a solid foothold in the health domain, where issues like regulation and acceptance demand increased attention. As the field continues to evolve, it is evident that AI will play an increasingly pivotal role in bridging various domains, and integrated ATs with AI are poised to assume a key role as a vital connector.

## Figures and Tables

**Table 1:** Key elements/points emerging from the overview of reviews on the intersection of AI and ATs.

Review Study	Key Points on the Intersection of AI and ATs in Autism
Muthu et al. [26]	Integration of AI in ATs for enhanced rehabilitation and independence.
Muthu et al. [26]	AI-driven solutions addressing physical impairments, mobility, education, and more.
Muthu et al. [26]	Insights into AI’s role in expanding research areas related to assistive technology.
Datta Barua et al. [27]	AI-assisted tools for improving learning and social interaction in neurodevelopmental disorders.
Datta Barua et al. [27]	Evidence supporting the effectiveness of AI tools in providing personalized education.
Alabdulkareem et al. [28]	Utilization of interactive robots with AI for autism therapy.
Alabdulkareem et al. [28]	Growth in research due to advancements in AI techniques and machine learning.
Ur Rehman et al. [29]	Identification of highly-rated mobile apps for individuals with ASD utilizing AI technologies.
Ur Rehman et al. [29]	Recommendations for enhancing existing applications with AI for personalized support.
Di Pietro et al. [30]	Exploration of AI-driven computer-assisted and robot-assisted therapies for children with autism.
Di Pietro et al. [30]	Focus on identifying AI platforms, professions involved, and outcomes in social skills teaching.
Den Brok et al. [31]	AI-powered self-controlled technologies aiding individuals with autism and intellectual disability.
Den Brok et al. [31]	Use of AI to facilitate the learning of daily living skills and cognitive concepts.
Billard et al. [32]	Application of AI in humanoid robots for assisting low-functioning children with autism.
Billard et al. [32]	AI’s role in assessing imitation ability and teaching coordinated behaviors.

**Table 2:** Key elements/points emerging from the overview of articles on the intersection of AI and ATs.

Article Study	Key Points on the Intersection of AI and ATs in Autism
Silvera Tawill et al. [33]	AI-driven socially-assistive robots for teaching support
Deng et al. [34]	AI-powered sensory management recommendation system for children with ASD.
Wan et al. [35]	AI-based system for improving emotion recognition in children with ASD.
Kumar et al. [36]	Automation of ASD diagnosis using machine learning techniques.
Jain et al. [37]	AI-driven models for recognizing and responding to user engagement in robot interventions.
Keshav et al. [38]	AI-driven models for recognizing and responding to user engagement in robot interventions.
Vahabzadeh et al. [39]	AI-driven smartglasses intervention for improving socio-emotional behaviors in students with ASD.
Cooper et al. [40]	AAC software program with an embedded artificial conversational agent for children with autism.
Huijnen et al. [41]	Roles, strengths, and challenges of AI-equipped robots in interventions for children with ASD.
Keshav et al. [42]	Tolerability and usability of AI-driven smartglasses for individuals with ASD.
Linstead et al. [43]	Usefulness in perspective of AI in the treatment dosage and in providing insights into its varied effects across different domains.
Desideri et al. [44]	Exploration of humanoid robots’ potential to enhance educational interventions for children with ASD.
Huijnen et al. [45]	Practical implementation of robots (implementing AI based algorithms), particularly robot KASPAR, in education and therapy interventions for children with ASD.
Bekele et al. [46]	Pilot study on an AI-driven robot-mediated system administering joint attention prompts to children with ASD with a demonstration of AI’s potential to enhance engagement and learning in educational activities for children with ASD.
Williams et al. [47]	Highlighting AI-like systems’ (with speech synthesizer) role in improving speech recognition and training for individuals with ASD.

## References

[1] https://www.cdc.gov/ncbddd/autism/facts.html.

[2] https://www.autismspeaks.org/what-autism.

[3] https://www.nhs.uk/conditions/autism/what-is-autism/.

[4] https://www.nimh.nih.gov/health/topics/autism-spectrum-disorders-asd.

[5] https://www.who.int/news-room/fact-sheets/detail/autism-spectrum-disorders.

[6] https://www.psychiatry.org/patients-families/autism/what-is-autism-spectrum-disorder.

[7] Mughal S., Faizy R.M., Saadabadi A. (2023). Autism Spectrum Disorder.

[8] Grabrucker A.M. (2021). Autims Spectrum disor$des Brisbane (AU).

[9] FM Casanova Imaging the Brain in Autism 2013th Edition, Springer 2013. https://www.amazon.it/Imaging-Brain-Autism-Manuel-Casanova/dp/1489999965.

[10] Liu M., Li B., Hu D. (2021). Autism Spectrum Disorder Studies Using fMRI Data and Machine Learning: A Review. Front Neurosci..

[11] ElNakieb Y., Ali M.T., Elnakib A., Shalaby A., Mahmoud A., Soliman A., Barnes G.N., El-Baz A. (2023). Understanding the Role of Connectivity Dynamics of Resting-State Functional MRI in the Diagnosis of Autism Spectrum Disorder: A Comprehensive Study. Bioengineering.

[12] Pruneti C., Coscioni G., Guidotti S. (2023). Evaluation of the effectiveness of behavioral interventions for autism spectrum disorders: A systematic review of randomized controlled trials and quasi-experimental studies. Clin. Child Psychol. Psychiatry.

[13] Hutchinson J., Folawemi O., Bittla P., Kaur S., Sojitra V., Zahra A., Khan S. (2023). The Effects of Risperidone on Cognition in People with Autism Spectrum Disorder: A Systematic Review. Cureus.

[14] Yu Z., Zhang P., Tao C., Lu L., Tang C. (2023). Efficacy of nonpharmacological interventions targeting social function in children and adults with autism spectrum disorder: A systematic review and meta-analysis. PLoS ONE..

[15] Watling R., Benevides T., Robertson S.M. (2023). Family-Centered Interventions for Children on the Autism Spectrum (2013–2021). Am. J. Occup Ther..

[16] Scarcella I., Marino F., Failla C., Doria G., Chilà P., Minutoli R., Vetrano N., Vagni D., Pignolo L., Di Cara M. (2023). Information and communication technologies-based interventions for children with autism spectrum conditions: A systematic review of randomized control trials from a positive technology perspective. Front. Psychiatry.

[17] https://www.nytimes.com/2022/03/29/technology/ai-robots-students-disabilities.html.

[18] Qiu S., An P., Kang K., Hu J., Han T., Rauterberg M. (2021). A Review of Data Gathering Methods for Evaluating Socially Assistive Systems. Sensors.

[19] Lima Antão J.Y.F., Oliveira A.S.B., Almeida Barbosa R.T., Crocetta T.B., Guarnieri R., Arab C., Massetti T., Antunes T.P.C., Silva A.P.D., Bezerra Ĺ.M.P. (2018). Instruments for augmentative and alternative communication for children with autism spectrum disorder: A systematic review. Clinics.

[20] Brignell A., Chenausky K.V., Song H., Zhu J., Suo C., Morgan A.T. (2018). Communication interventions for autism spectrum disorder in minimally verbal children. Cochrane Database Syst. Rev..

[21] Smith D.L., Atmatzidis K., Capogreco M., Lloyd-Randolfi D., Seman V. (2017). Evidence-Based Interventions for Increasing Work Participation for Persons with Various Disabilities. OTJR Occup. Particip. Health.

[22] https://www.verywellhealth.com/assistive-technology-for-autism-5076159.

[23] https://www.atandme.com/contribution-of-artificial-intelligence-on-assistive-technology-innovation/.

[24] ANDJ Checklist. https://legacyfileshare.elsevier.com/promis_misc/ANDJ%20Narrative%20Review%20Checklist.pdf.

[25] Giansanti D. (2022). The Regulation of Artificial Intelligence in Digital Radiology in the Scientific Literature: A Narrative Review of Reviews. Healthcare.

[26] Muthu P., Tan Y., Latha S., Dhanalakshmi S., Lai K.W., Wu X. (2023). Discernment on assistive technology for the care and support requirements of older adults and differently-abled individuals. Front. Public Health.

[27] Barua P.D., Vicnesh J., Gururajan R., Oh S.L., Palmer E., Azizan M.M., Kadri N.A., Acharya U.R. (2022). Artificial Intelligence Enabled Personalised Assistive Tools to Enhance Education of Children with Neurodevelopmental Disorders—A Review. Int. J. Environ. Res. Public Health.

[28] Alabdulkareem A., Alhakbani N., Al-Nafjan A. (2022). A Systematic Review of Research on Robot-Assisted Therapy for Children with Autism. Sensors.

[29] Rehman I.U., Sobnath D., Nasralla M.M., Winnett M., Anwar A., Asif W., Sherazi H.H.R. (2021). Features of Mobile Apps for People with Autism in a Post COVID-19 Scenario: Current Status and Recommendations for Apps Using AI. Diagnostics.

[30] DiPietro J., Kelemen A., Liang Y., Sik-Lanyi C. (2019). Computer- and Robot-Assisted Therapies to Aid Social and Intellectual Functioning of Children with Autism Spectrum Disorder. Medicina.

[31] den Brok W.L., Sterkenburg P.S. (2015). Self-controlled technologies to support skill attainment in persons with an autism spectrum disorder and/or an intellectual disability: A systematic literature review. Disabil. Rehabil. Assist. Technol..

[32] Billard A., Robins B., Nadel J., Dautenhahn K. (2007). Building Robota, a mini-humanoid robot for the rehabilitation of children with autism. Assist. Technol..

[33] Silvera-Tawil D., Bruck S., Xiao Y., Bradford D. (2022). Socially-Assistive Robots to Support Learning in Students on the Autism Spectrum: Investigating Educator Perspectives and a Pilot Trial of a Mobile Platform to Remove Barriers to Implementation. Sensors.

[34] Deng L., Rattadilok P. (2022). A Sensor and Machine Learning-Based Sensory Management Recommendation System for Children with Autism Spectrum Disorders. Sensors.

[35] Wan G., Deng F., Jiang Z., Song S., Hu D., Chen L., Wang H., Li M., Chen G., Yan T. (2022). FECTS: A Facial Emotion Cognition and Training System for Chinese Children with Autism Spectrum Disorder. Comput. Intell. Neurosci..

[36] Kumar C.J., Das P.R. (2021). The diagnosis of ASD using multiple machine learning techniques. Int. J. Dev. Disabil..

[37] Jain S., Thiagarajan B., Shi Z., Clabaugh C., Matarić M.J. (2020). Modeling engagement in long-term, in-home socially assistive robot interventions for children with autism spectrum disorders. Sci. Robot..

[38] Keshav N.U., Vogt-Lowell K., Vahabzadeh A., Sahin N.T. (2019). Digital Attention-Related Augmented-Reality Game: Significant Correlation between Student Game Performance and Validated Clinical Measures of Attention-Deficit/Hyperactivity Disorder (ADHD). Children.

[39] Vahabzadeh A., Keshav N.U., Abdus-Sabur R., Huey K., Liu R., Sahin N.T. (2018). Improved Socio-Emotional and Behavioral Functioning in Students with Autism Following School-Based Smartglasses Intervention: Multi-Stage Feasibility and Controlled Efficacy Study. Behav. Sci..

[40] Cooper A., Ireland D. (2018). Designing a Chat-Bot for Non-Verbal Children on the Autism Spectrum. Stud. Health Technol. Inform..

[41] Huijnen C.A.G.J., Lexis M.A.S., Jansens R., de Witte L.P. (2019). Roles, Strengths and Challenges of Using Robots in Interventions for Children with Autism Spectrum Disorder (ASD). J. Autism Dev. Disord..

[42] Keshav N.U., Salisbury J.P., Vahabzadeh A., Sahin N.T. (2017). Social Communication Coaching Smartglasses: Well Tolerated in a Diverse Sample of Children and Adults with Autism. JMIR Mhealth Uhealth.

[43] Linstead E., Dixon D.R., Hong E., Burns C.O., French R., Novack M.N., Granpeesheh D. (2017). An evaluation of the effects of intensity and duration on outcomes across treatment domains for children with autism spectrum disorder. Transl. Psychiatry.

[44] Desideri L., Negrini M., Cutrone M.C., Rouame A., Malavasi M., Hoogerwerf E.J., Bonifacci P., Di Sarro R. (2017). Exploring the Use of a Humanoid Robot to Engage Children with Autism Spectrum Disorder (ASD). Stud. Health Technol. Inform..

[45] Huijnen C.A.G.J., Lexis M.A.S., Jansens R., de Witte L.P. (2017). How to Implement Robots in Interventions for Children with Autism? A Co-creation Study Involving People with Autism, Parents and Professionals. J. Autism Dev. Disord..

[46] Bekele E., Crittendon J.A., Swanson A., Sarkar N., Warren Z.E. (2014). Pilot clinical application of an adaptive robotic system for young children with autism. Autism.

[47] Williams J.H., Massaro D.W., Peel N.J., Bosseler A., Suddendorf T. (2004). Visual-auditory integration during speech imitation in autism. Res. Dev. Disabil..

[48] https://www.cdc.gov/ncbddd/autism/screening.html#:~:text=Diagnosing%20autism%20spectrum%20disor-der%20(ASD,months%20of%20age%20or%20younger.

[49] https://www.cdc.gov/ncbddd/autism/hcp-dsm.html.

[50] Lord C., Elsabbagh M., Baird G., Veenstra-Vanderweele J. (2018). Autism spectrum disorder. Lancet.

[51] National Institute of Mental Health (2011). A Parent’s Guide to Autism Spectrum Disorder. http://www.nimh.nih.gov/health/publications/a-parents-guide-to-autism-spectrum-disorder/index.shtml.

[52] Kotte A., Joshi G., Fried R., Uchida M., Spencer A., Woodworth K.Y., Kenworthy T., Faraone S.V., Biederman J. (2013). Autistic traits in children with and without ADHD. Pediatrics.

[53] https://www.nichd.nih.gov/health/topics/autism/conditioninfo/treatments.

[54] https://www.who.int/news-room/fact-sheets/detail/assistive-technology.

[55] Goetz L.H., Schork N.J. (2018). Personalized medicine: Motivation, challenges, and progress. Fertil. Steril..

[56] Delpierre C., Lefèvre T. (2023). Precision and personalized medicine: What their current definition says and silences about the model of health they promote. Implication for the development of personalized health. Front. Sociol..

[57] Shlyakhto E.V. (2022). Scientific Basics of Personalized Medicine: Realities and Opportunities. Her. Russ. Acad. Sci..

[58] Evers A.W., Rovers M.M., Kremer J.A., Veltman J.A., Schalken J.A., Bloem B.R., van Gool A.J. (2012). An integrated framework of personalized medicine: From individual genomes to participatory health care. Croat. Med. J..

[59] Schork N.J. (2019). Artificial Intelligence and Personalized Medicine. Cancer Treat. Res..

[60] Johnson K.B., Wei W.Q., Weeraratne D., Frisse M.E., Misulis K., Rhee K., Zhao J., Snowdon J.L. (2021). Precision Medicine, AI, and the Future of Personalized Health Care. Clin. Transl. Sci..

[61] Loth E., Murphy D.G., Spooren W. (2016). Defining Precision Medicine Approaches to Autism Spectrum Disorders: Concepts and Challenges. Front. Psychiatry.

[62] Kostic A., Buxbaum J.D. (2021). The promise of precision medicine in autism. Neuron.

[63] Gabis L.V., Gross R., Barbaro J. (2021). Editorial: Personalized Precision Medicine in Autism Spectrum-Related Disorders. Front. Neurol..

[64] Frye R.E., Boles R., Rose S., Rossignol D. (2022). A Personalized Medicine Approach to the Diagnosis and Management of Autism Spectrum Disorder. J. Pers. Med..

[65] Kautish S., Dhiman G. (2021). Artificial Intelligence for Accurate Analysis and Detection of Autism Spectrum Disorder (Advances in Medical Diagnosis, Treatment, and Care).

[66] Mintz J., Gyori M., Aagaard M. (2012). Touching the Future Technology for Autism? Lessons from the HANDS Project.

[67] Wu X., Deng H., Jian S., Chen H., Li Q., Gong R., Wu J. (2023). Global trends and hotspots in the digital therapeutics of autism spectrum disorders: A bibliometric analysis from 2002 to 2022. Front. Psychiatry.

[68] Marciano F., Venutolo G., Ingenito C.M., Verbeni A., Terracciano C., Plunk E., Garaci F., Cavallo A., Fasano A. (2021). Artificial Intelligence: The “Trait D’Union” in Different Analysis Approaches of Autism Spectrum Disorder Studies. Curr. Med. Chem..

[69] Abdel Hameed M., Hassaballah M., Hosney M.E., Alqahtani A. (2022). An AI-Enabled Internet of Things Based Autism Care System for Improving Cognitive Ability of Children with Autism Spectrum Disorders. Comput. Intell. Neurosci..

[70] Giansanti D. (2023). An Umbrella Review of the Fusion of fMRI and AI in Autism. Diagnostics.

[71] Del Casale A., Sarli G., Bargagna P., Polidori L., Alcibiade A., Zoppi T., Borro M., Gentile G., Zocchi C., Ferracuti S. (2023). Machine Learning and Pharmacogenomics at the Time of Precision Psychiatry. Curr. Neuropharmacol..

[72] Ali S.G., Wang X., Li P., Jung Y., Bi L., Kim J., Chen Y., Feng D.D., Magnenat Thalmann N., Wang J. (2023). A systematic review: Virtual-reality-based techniques for human exercises and health improvement. Front. Public Health.

[73] Zhang S., Wang S., Liu R., Dong H., Zhang X., Tai X. (2022). A bibliometric analysis of research trends of artificial intelligence in the treatment of autistic spectrum disorders. Front. Psychiatry.

[74] Alqaysi M.E., Albahri A.S., Hamid R.A. (2022). Diagnosis-Based Hybridization of Multimedical Tests and Sociodemographic Characteristics of Autism Spectrum Disorder Using Artificial Intelligence and Machine Learning Techniques: A Systematic Review. Int. J. Telemed. Appl..

[75] Joudar S.S., Albahri A.S., Hamid R.A. (2022). Triage and priority-based healthcare diagnosis using artificial intelligence for autism spectrum disorder and gene contribution: A systematic review. Comput. Biol. Med..

[76] Welch V., Wy T.J., Ligezka A., Hassett L.C., Croarkin P.E., Athreya A.P., Romanowicz M. (2022). Use of Mobile and Wearable Artificial Intelligence in Child and Adolescent Psychiatry: Scoping Review. J. Med. Internet Res..

[77] Cavus N., Lawan A.A., Ibrahim Z., Dahiru A., Tahir S., Abdulrazak U.I., Hussaini A. (2021). A Systematic Literature Review on the Application of Machine-Learning Models in Behavioral Assessment of Autism Spectrum Disorder. J. Pers. Med..

[78] Quaak M., van de Mortel L., Thomas R.M., van Wingen G. (2021). Deep learning applications for the classification of psychiatric disorders using neuroimaging data: Systematic review and meta-analysis. Neuroimage Clin..

[79] Valencia K., Rusu C., Quiñones D., Jamet E. (2019). The Impact of Technology on People with Autism Spectrum Disorder: A Systematic Literature Review. Sensors.

[80] Valliani A.A., Ranti D., Oermann E.K. (2019). Deep Learning and Neurology: A Systematic Review. Neurol. Ther..

